# Distribution patterns and determinants of the lens thickness-to-anterior chamber depth ratio in cataract patients

**DOI:** 10.3389/fmed.2025.1654191

**Published:** 2025-10-08

**Authors:** Chunwen Zheng, Wenwen Geng, Ruirui Ma, Xiaoning Hao, Yuling Niu, Duanrong Cao, Yijun Hu, Ling Jin

**Affiliations:** ^1^Department of Ophthalmology, The People’s Hospital of Baoan Shenzhen, Shenzhen, China; ^2^Department of Ophthalmology, The Second Affiliated Hospital of Shenzhen University, Shenzhen, China; ^3^Department of Ophthalmology and Visual Sciences, The Chinese University of Hong Kong, Hong Kong, Hong Kong SAR, China; ^4^Department of Ophthalmology, Guangdong Eye Institute, Guangdong Provincial People’s Hospital (Guangdong Academy of Medical Sciences), Southern Medical University, Guangzhou, China

**Keywords:** LT/ACD, biometrics, determinants, cataract, cataract surgery

## Abstract

**Background:**

The lens thickness-to-anterior chamber depth (LT/ACD) ratio remains underexplored, despite its significance in optimizing cataract surgical outcomes and stratifying the risk of potential complications in aging populations.

**Aim:**

This study aimed to investigate the distribution patterns and determinants of the LT/ACD ratio in cataract patients.

**Methods:**

Bilateral ocular biometrics of 715 cataract patients were measured using Lenstar LS-900. The LT/ACD ratios of the right and left eyes were analyzed separately, with the results for the left eyes used to validate those for the right eyes. The LT/ACD ratio was compared using the Mann–Whitney or Kruskal–Wallis test. Spearman’s correlation coefficients were used to evaluate its correlation with other biometrics. Univariable and multivariable linear regression analyses were performed to identify the determinants of the LT/ACD ratio.

**Results:**

The LT/ACD ratio was higher in women, in patients with shorter axial length (AL), and in older patients (all *p* < 0.0001). In both eyes, the LT/ACD ratio correlated with iris center distance, pupil size (PS), angle kappa, AL, and white-to-white corneal diameter (WTW) (all *p* < 0.05). Determinants of the LT/ACD ratio in the right eyes included age (*β* = 0.01), sex (*β* = −0.08), anterior corneal astigmatism (ACA) (*β* = 0.06), angle kappa (*β* = 0.30), and AL (*β* = −0.09). In the left eyes, determinants included age (*β* = 0.01), sex (*β* = −0.08), corneal curvature (CR) (*β* = −0.05), angle kappa (*β* = 0.20), AL (*β* = −0.12), and WTW (*β* = −0.12).

**Conclusion:**

The distribution patterns of the LT/ACD ratio varied with sex, AL, and age, and the LT/ACD ratio correlated with similar but distinct determinants in both eyes. These findings help us better understand the interaction between LT and ACD in the eyes of cataract patients.

## Introduction

As a leading cause of reversible blindness worldwide, cataracts affect over 79 million individuals older than 50 years old, with many ensuing ophthalmic complications that pose a significant burden on healthcare systems (1–3). With the advancements in techniques, cataract surgery has evolved beyond a vision-restorative intervention to a precision-driven refractive procedure and has even expanded its role in preventing other sight-threatening complications (1, 4, 5). All of these improvements require accurate prediction of intraocular lens (IOL) position and postoperative refractive outcomes, which rely heavily on comprehensive ocular biometric measurements (6).

Among key ocular biometrics, lens thickness (LT) and anterior chamber depth (ACD) are two basic yet critical parameters derived from the anterior segment of the eye in the axial dimension, and they are used to assess the anterior segment dynamics and assist in surgical risk stratifications (7, 8). Notably, LT is known to increase with age, which is a key factor in the development of senile cataracts (9). Previous epidemiological studies reported that a thicker LT or shallower ACD was associated with a narrower anterior chamber angle, which is one of the determinants in the development and progression of angle-closure glaucoma (10–12). However, LT and ACD were indicated to be non-independently associated with each other by a population-based study, indicating the complex interplay between them (12–14). For example, during the gradual thickening of the lens in senile cataract, a thicker lens is usually followed by a deeper anterior chamber (8, 15). The ratio of LT to ACD (LT/ACD) has emerged as a clinically significant biomarker. The LT/ACD ratio considers the spatial relationship between the crystalline lens and the anterior chamber, offering insights into anterior segment dynamics. Its important clinical indications include refining the effective lens position (ELP) estimation of IOL in cataract surgery, assessing zonular stability, and identifying eyes with an anatomical predisposition to angle closure (16).

Although LT and ACD have been studied and highlighted individually in many studies, the LT/ACD ratio remains underexplored, particularly in relation to bilateral ocular symmetry, demographic factors, and the correlations between other ocular biometrics (16). In aging populations, progressive lens thickening and anterior chamber shallowing elevate the LT/ACD ratio synergistically, predisposing eyes to angle closure, which is a key mechanism underlying angle-closure glaucoma (17, 18). This is particularly critical in Asian populations with cataracts, where an increased LT/ACD ratio exceeding 1.8 has been reported to effectively identify anterior chamber angle narrowness, indicating that the LT/ACD ratio can serve as a strong ocular biometric determinant of potential angle-closure glaucoma (16). Despite its clinical relevance, the distribution patterns and determinants of the LT/ACD ratio in patients with cataracts remain poorly characterized, particularly between bilateral eyes and across different gender, age, and axial length (AL) groups. Furthermore, the interactions between the LT/ACD ratio and other biometrics known to affect both glaucoma and refractive outcomes have not been explored. These research gaps limit personalized surgical planning and potential glaucoma risk prediction.

This study aimed to address these gaps by investigating the distribution patterns and determinants of the LT/ACD ratio in patients with cataracts using ocular biometric measurements. By identifying the biometric correlates and validating the interocular consistency, our study aimed to provide a framework for refining glaucoma screening protocols, personalizing IOL formulas, and tailoring surgical interventions in phakic eyes with cataracts of high-risk glaucoma.

## Methods

### Study design and participants

In this retrospective, cross-sectional study, 715 cataract patients who underwent biometric measurements at the People’s Hospital of Baoan Shenzhen (Shenzhen, China) between 2022 and 2024 were recruited. The inclusion criteria were as follows: (1) patients aged 45 years or older; (2) those with a diagnosis of cataract by our ophthalmologists; and (3) those with the ability to cooperate effectively with the biometric measurements in both eyes. The exclusion criteria were as follows: (1) a previous medical history of ocular surgery, eye trauma, ocular surface, or corneal abnormalities; (2) patients with a diagnosis of vitreous or retinal diseases; (3) those with a diagnosis of glaucoma; (4) those with a diagnosis of pseudoexfoliation syndrome; (5) those diagnosed with aphakic and pseudophakic conditions; and (6) those with any other ocular conditions that could affect measurement results. This study was conducted in adherence to the Declaration of Helsinki, and the study was approved by the Institutional Review Board (IRB) of the People’s Hospital of Baoan, Shenzhen (BYL20240638). Written informed consent was waived because no participants could be identified from the data.

The right and left eyes of the recruited patients were divided into two sex groups (female patients and male patients), three axial length groups (group 1: AL ≤ 22 mm; group 2: 22 mm < AL < 25 mm; group 3: AL ≥ 25 mm), and four age groups (45–54 years old, 55–64 years old, 65–74 years old, and ≥75 years old).

### Ocular biometrics

**Figure 1:**
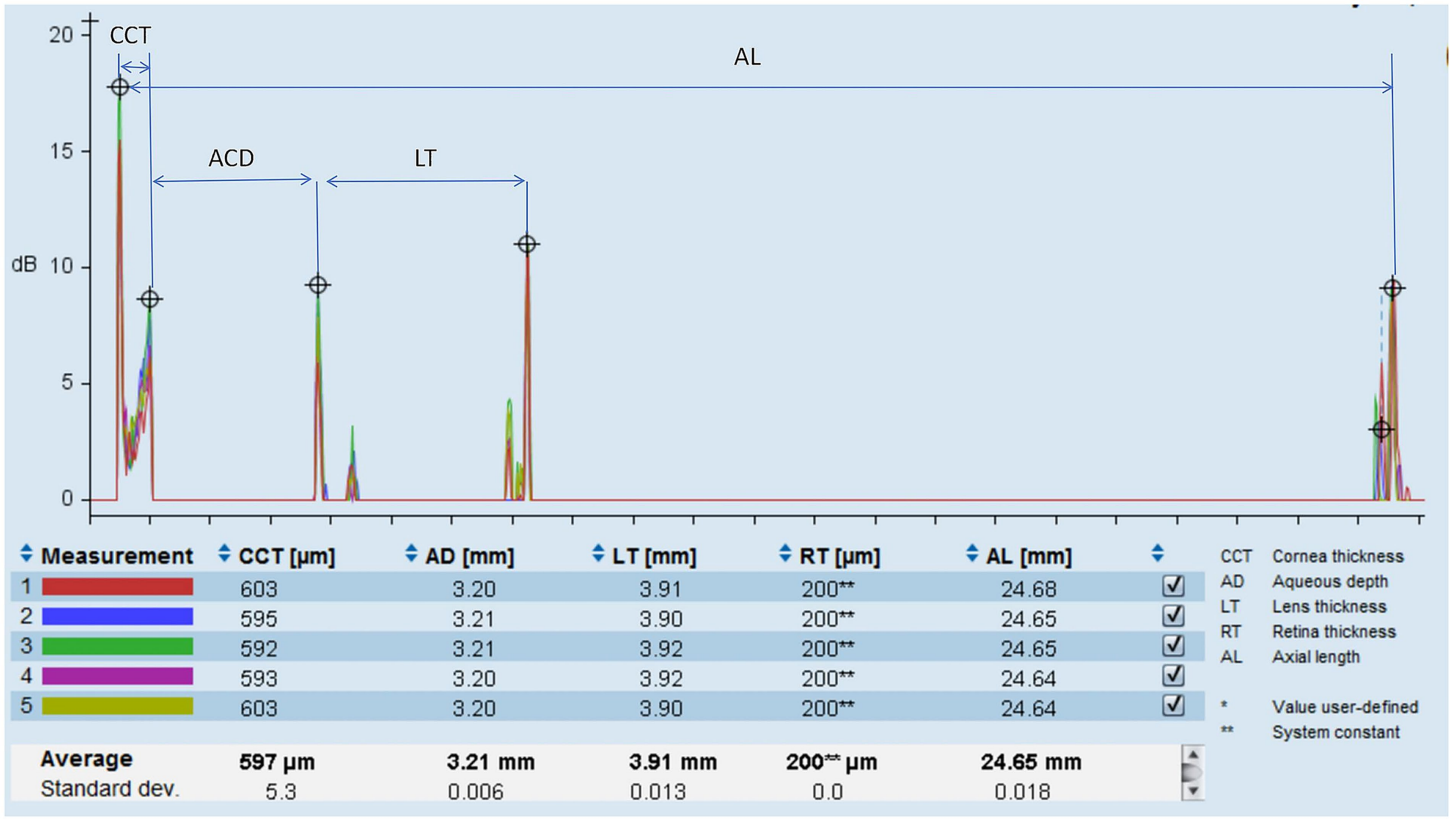
Ocular biometric measurements interface on Lenstar LS-900.

Ocular biometrics were measured using optical low-coherence reflectometry (Lenstar LS-900, Haag-Streit AG, Switzerland) (Figure 1). Bilateral ocular biometric measurements of eligible patients were performed by certified technicians at our hospital. Patients were instructed to focus on the cursor displayed on the device under standard illumination conditions until the bilateral ocular measurements were obtained at one sitting. Any low-quality results were excluded and remeasured accordingly. The biometrics measured by the Lenstar LS-900 included LT, ACD, central corneal thickness (CCT), corneal curvature (CR), anterior corneal astigmatism (ACA), iris center distance (distance from the centroid of the iris to the center of the cornea), pupil size (PS), angle kappa (angle between the visual and pupillary axes), axial length (AL), and white-to-white corneal diameter (WTW). At least three consecutive measurements per parameter were averaged. LT was defined as the distance from the anterior to the posterior surfaces of the crystalline lens along the visual axis. ACD was determined as the distance from the anterior cornea to the anterior lens surface on the visual axis. The LT/ACD ratio was defined and calculated as the LT divided by the ACD.

### Statistical analysis

We performed the analyses with a rigorous separation of the two eyes to account for potential interocular correlations. The LT/ACD ratios of the right and left eyes were also analyzed separately, with the results of the left eyes used to validate those of the right eyes. Normality was assessed using Shapiro–Wilk tests, and non-parametric tests were applied for skewed data. Medians of the LT/ACD ratio in different groups were compared using the Mann–Whitney *U*-tests (sex group) or the Kruskal–Wallis test (ALs or age groups) with Bonferroni correction. Spearman’s correlation coefficients with a 95% confidence interval (CI) were used to evaluate the correlations between the LT/ACD ratio and other biometrics, including CCT, CR, ACA, iris center distance, PS, angle kappa, AL, and WTW. A univariable linear regression analysis was preliminarily performed to identify candidate covariates of the LT/ACD ratio. Then, a multivariable linear regression analysis was performed after adjusting for significant covariates and used to identify determinants of the LT/ACD ratio. In the multivariable model, age, sex, ACA, iris center distance, PS, angle kappa, AL, and WTW were included as the independent variables, while the LT/ACD ratios in the right and left eyes were the dependent variables. Model collinearity was assessed using variance inflation factors (VIFs), and any independent variable with a VIF of >10 was eliminated from the model. Regression coefficients (*β*) and 95% confidence intervals (CIs) were reported. SPSS Statistics was used (version 26.0; IBM Corporation, SPSS Inc., Chicago, IL, United States) for statistical analysis; *p* < 0.05 was considered to be statistically significant.

## Results

### Distribution patterns of the LT/ACD ratio across different demographic groups

There were 715 patients (323 men and 392 women) included in our study, with a mean age of 65.6 years. The distribution patterns of the LT/ACD ratio showed significant variations across different sex, ages, and AL groups in patients with cataracts (all *p* < 0.05).

The LT/ACD ratio showed significant differences among patients of different sexes (Table 1). The median [interquartile range (IQR): 25th percentile and 75th percentile] of the LT/ACD ratio was significantly higher in women than in men, with LT/ACD = 1.88 (1.59, 2.24) for women and 1.70 (1.47, 2.01) for men in the right eyes (*p* < 0.0001). Similar results were shown in the left eyes, with LT/ACD = 1.93 (1.60, 2.26) in women and 1.70 (1.47, 2.07) in men (*p* < 0.0001). Analysis of the LT/ACD in different AL groups revealed a significant inverse correlation between the LT/ACD ratios and AL (Table 1). The eyes in group 1 had a higher LT/ACD ratio [LT/ACD = 2.39 (1.91, 2.67) in the right eyes; LT/ACD = 2.22 (1.99, 2.70) in the left eyes] than those in group 2 [LT/ACD = 1.83 (1.57, 2.18) in the right eyes; LT/ACD = 1.86 (1.59, 2.20) in the left eyes] or group 3 [LT/ACD = 1.46 (1.30, 1.62) in the right eyes; LT/ACD = 1.47 (1.33, 1.68) in the left eyes] (*p* < 0.0001).

**Table 1:** Distribution of the LT/ACD ratio by sex and AL groups^a^.

	Right eye	Left eye	*p*
Sex groups			
Male patients	1.70 (1.47, 2.01)	1.70 (1.47, 2.07)	**0.006**
Female patients	1.88 (1.59, 2.24)	1.93 (1.60, 2.26)	**0.031**
*p*	**<0.0001**	**<0.0001**	
*U*-value	76845.5	75,643	
AL groups			
AL ≤ 22 mm	2.39 (1.91, 2.67)	2.22 (1.99, 2.70)	0.974
22 mm < AL < 25 mm	1.83 (1.57, 2.18)	1.86 (1.59, 2.20)	0.305
AL ≥ 25 mm	1.46 (1.30, 1.62)	1.47 (1.33, 1.68)	0.545
*p*	**<0.0001**	**<0.0001**	
*χ* ^2^	110.35	100.58	
df	2	2	
*η* ^2^	0.15	0.14	

LT/ACD ratio, lens thickness-to-anterior chamber depth ratio; AL, axial length; *U*, *U* value of the Mann–Whitney *U*-tests; *χ*^2^ value, df (degrees of freedom), and *η*^2^ (effect size) are from the Kruskal–Wallis tests. Bold values indicate statistical significance (*p* < 0.05).

a Presented as median [interquartile range (IQR) 25th percentile, 75th percentile].

Additionally, analysis of the LT/ACD ratio in different age groups further demonstrated a progressive increase in the LT/ACD ratio with advancing age, increasing from 1.53 (1.34, 1.77) in the 45–54-year-old group to 2.10 (1.77, 2.35) in patients ≥75 years old in the right eyes (*p* < 0.0001) (Table 2). Similar findings were found in the left eyes, where the LT/ACD ratio increased progressively with older age from 1.53 (1.35, 1.76) to 2.10 (1.82, 2.38) (*p* < 0.0001). Interestingly, interocular differences in LT/ACD ratios were significant in the 65–74-year-old and ≥75-year-old groups, highlighting the compounding effects of age-related lens thickening and anterior chamber shallowing.

**Table 2:** Distribution of the LT/ACD ratio by different age groups^a^.

	Right eye	Left eye	*p*
Age groups			
45–54 years old	1.53 (1.34, 1.77)	1.53 (1.35, 1.76)	0.707
55–64 years old	1.68 (1.48, 1.97)	1.67 (1.47, 2.05)	0.064
65–74 years old	1.88 (1.65, 2.23)	1.95 (1.68, 2.26)	**0.040**
≥75 years old	2.10 (1.77, 2.35)	2.10 (1.82, 2.38)	**0.025**
*p*	**<0.0001**	**<0.0001**	
*χ* ^2^	103.51	110.38	
df	3	3	
*η* ^2^	0.14	0.15	

LT/ACD ratio, lens thickness-to-anterior chamber depth ratio; AL, axial length; *χ*^2^ value, df (degrees of freedom), and *η*^2^ (effect size) are from the Kruskal–Wallis tests. Bold values indicate statistical significance (*p* < 0.05).

a Presented as median (interquartile range [IQR] 25th percentile, 75th percentile).

### Correlations between the LT/ACD ratio and other ocular biometrics

**Figure 2:**
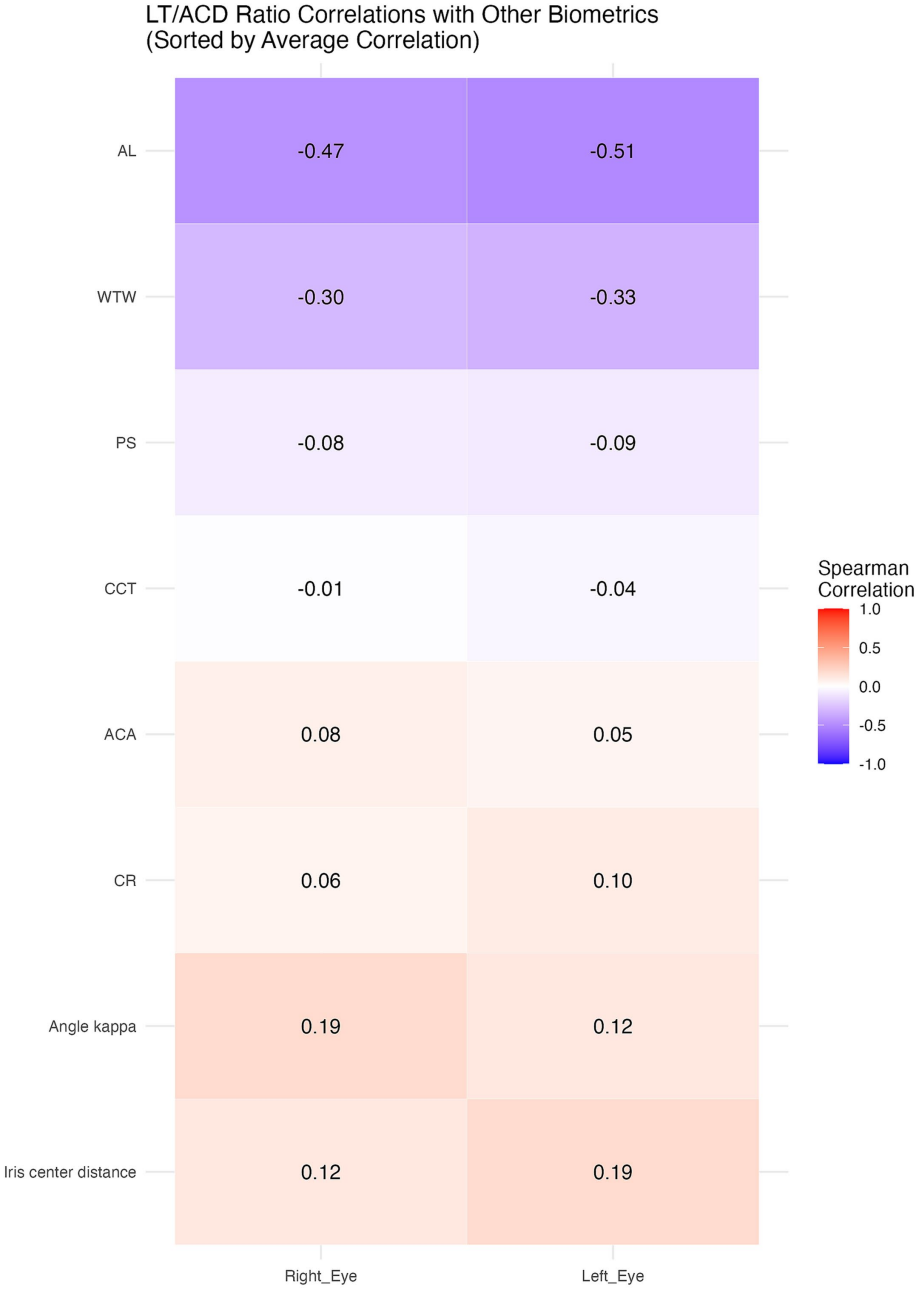
Heatmap demonstrating the correlations.

Significant correlations between the LT/ACD ratio and certain anterior segment biometrics are shown in Table 3, with notable interocular variability. A heatmap was also used to better demonstrate the correlations (Figure 2).

**Table 3:** Correlations between the LT/ACD ratio and other biometrics^a^.

Biometrics	Right eye	Left eye
CCT	−0.01 (−0.08 to 0.07)	−0.04 (−0.11 to 0.04)
CR	0.06 (−0.02 to 0.13)	**0.10 (0.02 to 0.17)**
ACA	**0.08 (0.002 to 0.15)**	0.05 (−0.02 to 0.13)
Iris center distance	**0.12 (0.04 to 0.19)**	**0.19 (0.12 to 0.26)**
PS	**−0.08 (−0.15 to −0.0003)**	**−0.09 (−0.16 to −0.01)**
Angle kappa	**0.19 (0.12 to 0.26)**	**0.13 (0.05 to 0.20)**
AL	**−0.47 (−0.53 to −0.41)**	**−0.51 (−0.56 to −0.45)**
WTW	**−0.30 (−0.36 to −0.23)**	**−0.33 (−0.39 to −0.26)**

Bold values indicate statistical significance (*p* < 0.05); LT/ACD ratio, lens thickness-to-anterior chamber depth ratio; CCT, central corneal thickness; CR, corneal curvature; ACA, anterior corneal astigmatism; PS, pupil size; AL, axial length; WTW, white-to-white corneal diameter.

a Presented as Spearman’s correlation coefficients (*R*, 95% confidence interval).

In both the right and left eyes, the LT/ACD ratio was found to be negatively correlated with PS (*R* = −0.08, 95% CI: −0.15 to −0.0003 in the right eyes; *R* = −0.09, 95% CI: −0.16 to −0.01 in the left eyes), AL (*R* = −0.47, 95% CI: −0.53 to −0.41 in the right eyes; *R* = −0.51, 95% CI: −0.56 to −0.45 in the left eyes), and WTW (*R* = −0.30, 95% CI: −0.36 to −0.23 in the right eyes; *R* = −0.33, 95% CI: −0.39 to −0.26 in the left eyes) (*p* < 0.05). Meanwhile, positive correlations were identified between the LT/ACD ratio and iris center distance (*R* = 0.12, 95% CI: 0.04 to 0.19 in the right eyes; *R* = 0.19, 95% CI: 0.12 to 0.26 in the left eyes), and angle kappa (*R* = 0.19, 95% CI: 0.12 to 0.26 in the right eyes; *R* = 0.13, 95% CI: 0.05 to 0.20 in the left eyes) in both eyes (*p* < 0.05). Notably, ACA was only significantly correlated with the LT/ACD ratio in the right eyes (*R* = 0.08, 95% CI: 0.002 to 0.15) (*p* < 0.05), while CR showed significance solely in the left eyes (*R* = 0.10, 95% CI: 0.02 to 0.17) (*p* < 0.05), suggesting interocular variability in anterior segment biometrics.

### Determinants of the LT/ACD ratio among ocular biometrics

The univariable linear regression analysis was first performed on the ocular biometrics to identify candidate covariates of the LT/ACD ratio (Table 4). A multivariable linear regression analysis was then performed, adjusting for the significant covariates, to identify determinants of the LT/ACD ratio (Table 5). In the multivariable model, age, sex, ACA, iris center distance, PS, angle kappa, AL, and WTW were used as the independent variables.

**Table 4:** Determinants of the LT/ACD ratio in a univariable linear regression analysis.

Determinants	Right eye		Left eye	
	Beta	95% CI	Beta	95% CI
Age	**0.02**	**0.01 to 0.02**	**0.02**	**0.01 to 0.02**
Sex	**−0.15**	**−0.22 to −0.09**	**−0.14**	**−0.21 to −0.07**
CCT	0.00	0.00 to 0.00	0.00	0.00 to 0.00
CR	0.02	−0.01 to 0.04	**0.03**	**0.004 to 0.05**
ACA	**0.07**	**0.02 to 0.11**	0.04	0.00 to 0.08
Iris center distance	**0.17**	**0.03 to 0.31**	**0.36**	**0.22 to 0.50**
PS	**−0.02**	**−0.05 to −0.0002**	−0.02	−0.05 to 0.00
Angle kappa	**0.46**	**0.26 to 0.66**	**0.25**	**0.06 to 0.43**
AL	**−0.12**	**−0.14 to −0.10**	**−0.14**	**−0.16 to −0.12**
WTW	**−0.21**	**−0.26 to −0.15**	**−0.23**	**−0.28 to −0.17**

CI, confidence interval; bold values indicate statistical significance (*p* < 0.05), lens thickness-to-anterior chamber depth ratio; CCT, central corneal thickness; CR, corneal curvature; ACA, anterior corneal astigmatism; PS, pupil size; AL, axial length; WTW, white-to-white corneal diameter.

**Table 5:** Determinants of the LT/ACD ratio in a multivariable linear regression analysis.

Determinants	Right eye	Left eye		
	Beta	95% CI	Beta	95% CI
Age	**0.01**	**0.01 to 0.02**	**0.01**	**0.01 to 0.01**
Sex	**−0.08**	**−0.14 to −0.02**	**−0.08**	**−0.14 to −0.02**
CCT	—	—	—	—
CR	—	—	**−0.05**	**−0.07 to −0.03**
ACA	**0.06**	**0.01 to 0.10**	—	—
Iris center distance	−0.12	−0.25 to 0.01	−0.02	−0.15 to 0.11
PS	−0.01	−0.03 to 0.01	—	—
Angle kappa	**0.30**	**0.11 to 0.49**	**0.20**	**0.04 to 0.37**
AL	**−0.09**	**−0.12 to −0.07**	**−0.12**	**−0.14 to −0.10**
WTW	−0.04	−0.10 to 0.02	**−0.12**	**−0.18 to −0.06**

CI, confidence interval; bold values indicate statistical significance (*p* < 0.05); LT/ACD ratio, lens thickness-to-anterior chamber depth ratio; CCT, central corneal thickness; CR, corneal curvature; ACA, anterior corneal astigmatism; PS, pupil size; AL, axial length; WTW, white-to-white corneal diameter.

In the univariable regression analysis, age, sex, iris center distance, angle kappa, AL, and WTW were reported as significant predictors of the LT/ACD ratio in both eyes (all *p* < 0.05). While ACA and PS were solely significant in the right eyes, and CR was solely significant in the left eyes (all *p* < 0.05). Multivariable regression analyses revealed divergent determinants of the LT/ACD ratio between the eyes. For the right eyes, age (*β* = 0.01/year, 95% CI: 0.01 to 0.02), female sex (*β* = −0.08, 95% CI: −0.14 to −0.02), ACA (*β* = 0.06, 95% CI: 0.01 to 0.10), angle kappa (*β* = 0.30, 95% CI: 0.11 to 0.49), and AL (*β* = −0.09, 95% CI: −0.12 to −0.07) were found as significant determinants of the LT/ACD ratio (all *p* < 0.05). For the left eyes, determinants of the LT/ACD ratio included age (*β* = 0.01/year, 95% CI: 0.01 to 0.01), female sex (*β* = −0.08, 95% CI: −0.14 to −0.02), CR (*β* = −0.05, 95% CI: −0.07 to −0.03), angle kappa (*β* = 0.20, 95% CI: 0.04 to 0.37), AL (*β* = −0.12, 95% CI: −0.14 to −0.10), and WTW (*β* = −0.12, 95% CI: −0.18 to −0.06) (all *p* < 0.05). Among these significant determinants, angle kappa was the strongest positive factor in both the right and left eyes, while AL and WTW had the strongest negative effect in the left eyes.

## Discussion

A meticulous ocular biometric evaluation is important for precise cataract surgery and the proactive management of surgical complications (19, 20). This study analyzed bilateral ocular biometrics from 715 patients with cataracts, underscoring the clinical significance of the LT/ACD ratio in cataracts. Our findings revealed significant variations in the LT/ACD ratio across different demographic subgroups and identified distinct biometric determinants for both right and left eyes of patients with cataracts, aiming to optimize cataract surgery outcomes and risk stratification for potential glaucoma, particularly in aging populations.

In our study, the demographic variations in the LT/ACD ratio were highlighted, with higher ratios observed in women, older adults, and eyes with shorter ALs. For example, women demonstrated a median LT/ACD ratio of 1.88 compared to 1.70 in men in the right eyes, reflecting sex-specific anatomical differences such as shorter ALs and shallower anterior chambers, which are established risk factors for angle closure. While the age-specific analysis revealed a progressive increase in the LT/ACD ratio from 1.53 in the 45–54-year-old group to 2.10 in the ≥75-year-old group in bilateral eyes, indicating the age-related lens thickening and anterior chamber shallowing, this progressive increase is consistent with the natural history of cataract formation, characterized by age-related lens thickening (9). These anatomical changes are not only relevant to cataract development but are also established risk factors for angle closure. Furthermore, the high prevalence of myopia in Asian populations is reflected in our cohort, and our analysis by AL groups captures its significant inverse relationship with the LT/ACD ratio (21). A short AL (≤22 mm) showed markedly higher LT/ACD ratios, underscoring the interplay between AL and shallowing of the anterior chamber. Additionally, the LT/ACD ratio demonstrated distinct correlations with various anterior segment biometric parameters, including angle kappa, AL, and WTW, with notable interocular variability. These results highlight the LT/ACD ratio as a clinically relevant biomarker for cataract surgery planning and glaucoma risk stratification.

The LT/ACD ratio integrates two important biometrics of the anterior segment, offering a novel dynamic and mutual assessment of the spatial relationship between the lens and anterior chamber (16). Its clinical significance was particularly emphasized in populations at high risk of angle closure, such as the Asian population with cataracts. In 2025, Miao et al. (16) identified an LT/ACD ratio threshold of 1.8 as a robust indicator of anterior chamber angle narrowness with an anterior chamber angle width of <20 degrees. Consistent with this prior study conducted in the Asian population, our findings reinforce the threshold that women, older adults, and individuals with shorter ALs have a heightened susceptibility to angle closure (11, 12, 22). Furthermore, the negative correlation between the LT/ACD ratio and AL aligns with the biomechanical evidence that shorter eyes predispose to anterior segment shallowing (23, 24). The multivariable regression analysis further investigated the determinants of the LT/ACD ratio, where age, sex, angle kappa, and AL exerted significant effects in bilateral eyes. These findings are consistent with the natural history of senile cataracts characterized by progressive lens thickening, sex disparities due to hormonal and anatomical differences, and the close relevance of angle kappa and AL in postoperative refractive outcomes of cataract surgery (8, 14, 15, 18).

Significant interocular differences were also found in our study. This asymmetry highlights the importance of performing careful biometric measurements on each eye individually, even in patients with bilateral cataracts, and not relying on the assumption of perfect symmetry. Interocular variability in determinants, such as CR, ACA, and WTW, suggests anatomical asymmetries and the necessity of eye-specific surgical planning, further validating the need for individualized approaches. In terms of surgical planning, the interocular differences of the LT/ACD ratio have important clinical implications. The LT/ACD ratio is a component of the latest generations of IOL power formulas that aim to predict the ELP more accurately. Significant interocular differences in the LT/ACD ratio imply that the ELP may also differ between eyes. Therefore, using biometric measurements from each eye independently is crucial for achieving the target refractive outcome in both eyes and minimizing postoperative anisometropia. Since the LT/ACD ratio is a known index for angle closure risk, an eye with a significantly higher ratio may warrant a more detailed preoperative gonioscopy. This helps ophthalmologists anticipate potential intraoperative risks that are more common in eyes with a shallow anterior chamber. In addition, recognizing anatomical asymmetry allows ophthalmologists to better counsel patients about the potential for differing visual and refractive outcomes between the eyes.

During the analysis of the distribution patterns and determinants of the LT/ACD ratio in patients with cataracts, our study has both strengths and limitations. Our study was conducted in a large sample population with bilateral ocular biometric measurement prior to cataract surgery. A novel ocular biometric index, the LT/ACD ratio, was introduced to show the interplay among ocular biometrics and provide insights into its importance in future clinical implementation. However, limitations in our study also warrant consideration. First, the retrospective study design failed to assess the causal inferences between the LT/ACD ratio and other ocular biometrics of the anterior segment. Second, our study recruited patients from China, which may affect regional sampling bias and limit the generalizability of the findings to non-Asian populations. Moreover, other important dynamic biomechanical parameters, such as intraocular pressure, which also affects ACD, were not analyzed, and more ocular parameters should also be considered (25). Furthermore, data on systemic comorbidities (e.g., diabetes) and certain ocular comorbidities (e.g., glaucoma, pseudoexfoliation syndrome) known to influence ocular biometrics were not systematically included or available for analysis (26). Their potential influences on ocular biometrics could not be accounted for, which may limit the generalizability of our findings. Future longitudinal studies should explore the predictive value of the LT/ACD ratio for postoperative outcomes and glaucoma progression in multi-ethnic populations. Additionally, cataract grading based on lens opacity (e.g., the LOCS III system) was not consistently available for analysis. As a higher grade of cataract is associated with increased lens thickness, its omission is a limitation, and future prospective studies should incorporate cataract grading to better elucidate its specific contribution to the LT/ACD ratio. Further research to explore its utility in phakic eyes without cataracts is also warranted.

## Conclusion

This retrospective, cross-sectional study revealed distinct distribution patterns of the LT/ACD ratio according to sex, AL, and age, and various determinants of the LT/ACD ratio in both eyes. Our findings highlight the significance of the LT/ACD ratio as a novel and critical ocular biometric index for angle-closure glaucoma risk assessment and cataract surgery optimization. By incorporating this index into clinical practice, it is likely to enhance the screening accuracy of glaucoma, refine the refractive predictability for the IOL calculations, and assess the anatomical asymmetries in aging eyes.

## Data Availability

The raw data supporting the conclusions of this article will be made available by the authors, without undue reservation.
